# Suitability of 3D human brain spheroid models to distinguish toxic effects of gold and poly-lactic acid nanoparticles to assess biocompatibility for brain drug delivery

**DOI:** 10.1186/s12989-019-0307-3

**Published:** 2019-06-03

**Authors:** Paulo Emílio Corrêa Leite, Mariana Rodrigues Pereira, Georgina Harris, David Pamies, Lisia Maria Gobbo dos Santos, José Mauro Granjeiro, Helena T. Hogberg, Thomas Hartung, Lena Smirnova

**Affiliations:** 1 Directory of Metrology Applied to Life Sciences – Dimav, National Institute of Metrology Quality and Technology – INMETRO, Av. Nossa Senhora das Graças 50, LABET - Dimav, Predio 27, Duque de Caxias, Xerem, Rio de Janeiro 25250-020 Brazil; 2 Biology Institute, Fluminense Federal University, Niteroi, Rio de Janeiro Brazil; 3 Center for Alternatives to Animal Testing (CAAT), Bloomberg School of Public Health, Johns Hopkins University, 615 N. Wolfe Street, Baltimore, MD 21205 USA; 4 Department of Chemistry, National Institute of Quality Control in Health – INCQS/Fiocruz, Manguinhos, Rio de Janeiro 21040-900 Brazil; 5 University of Konstanz, Biology, Konstanz, Germany; 6 Dental School, Fluminense Federal University, Niteroi, Rio de Janeiro USA; 7 Department of Physiology, University of Lausanne, Lausanne, CH-1015 USA

**Keywords:** Nanoparticle, Drug delivery, CNS, iPSC-derived BrainSpheres, 3D LUHMES

## Abstract

**Background:**

The blood brain barrier (BBB) is the bottleneck of brain-targeted drug development. Due to their physico-chemical properties, nanoparticles (NP) can cross the BBB and accumulate in different areas of the central nervous system (CNS), thus are potential tools to carry drugs and treat brain disorders. In vitro systems and animal models have demonstrated that some NP types promote neurotoxic effects such as neuroinflammation and neurodegeneration in the CNS. Thus, risk assessment of the NP is required, but current 2D cell cultures fail to mimic complex in vivo cellular interactions, while animal models do not necessarily reflect human effects due to physiological and species differences.

**Results:**

We evaluated the suitability of in vitro models that mimic the human CNS physiology, studying the effects of metallic gold NP (AuNP) functionalized with sodium citrate (Au-SC), or polyethylene glycol (Au-PEG), and polymeric polylactic acid NP (PLA-NP). Two different 3D neural models were used (i) human dopaminergic neurons differentiated from the LUHMES cell line (3D LUHMES) and (ii) human iPSC-derived brain spheroids (BrainSpheres). We evaluated NP uptake, mitochondrial membrane potential, viability, morphology, secretion of cytokines, chemokines and growth factors, and expression of genes related to ROS regulation after 24 and 72 h exposures. NP were efficiently taken up by spheroids, especially when PEGylated and in presence of glia. AuNP, especially PEGylated AuNP, effected mitochondria and anti-oxidative defense. PLA-NP were slightly cytotoxic to 3D LUHMES with no effects to BrainSpheres.

**Conclusions:**

3D brain models, both monocellular and multicellular are useful in studying NP neurotoxicity and can help identify how specific cell types of CNS are affected by NP.

**Electronic supplementary material:**

The online version of this article (10.1186/s12989-019-0307-3) contains supplementary material, which is available to authorized users.

## Background

Nanoparticles (NP) can be synthesized from different materials and have generated increasing interest in nanomedicine, mostly as drug delivery systems [1, 2]. NP capable of delivering drugs exhibit advantages such as drug stability, increased bioavailability and reduced drug concentrations required to reach the target, thus decreasing side effects [3]. Some NP, including gold NP, have the ability to cross the blood brain barrier (BBB) and reach the central nervous system (CNS), providing promising drug delivery systems (especially for the treatment of CNS diseases [3–8]). NP surface modification by adding specific targeting ligands can enhance their BBB penetration and contribute for cellular uptake. NP size, shape, surface, charge and modification with cell-penetration peptides matter for cellular uptake and fate. Commonly, smaller NPs are better internalized with higher percentage of biodistribution [9–11] and display more ability for BBB penetration and distribution in the CNS [12, 13]. Positively charged NPs are more easily internalized than neutral and negatively NPs [14]. In this sense, NP may serve as an important alternative for invasive CNS procedures such as implantation of catheters, imaging and therapy of brain tumors, and improvement of drug delivery [3, 15–17].

However, toxic effects of nanomaterials have been described [1], including induction of oxidative stress, inflammation [18], DNA damage, and alterations in gene expression [19]. We found earlier, for example, that cobalt nanoparticles were able to induce cell transformation, a hallmark of cancer, in contrast to cobalt ions [20]. NP can also promote neurotoxic effects such as neuroinflammation and neurodegeneration [21]. Despite gold NP (Au-NP) being one of the mostly commonly used nanomaterials (due to their versatility in particle size and surface modification) and their biocompatibility for applications such as drug and gene delivery [22], they can induce toxicity in different cellular models [23]. In addition, studies have shown that Au-NP induce apoptosis via caspase-dependent mechanisms as well as increase cell susceptibility to apoptosis induced by other agents [24, 25].

Polylactic acid NP (PLA-NP) is widely used as an alternative for drug delivery, including CNS. PLA-NP allow sustained therapeutic drug levels for longer periods due to their polymeric matrix that prevents drug degradation, allowing better release kinetics. The surface functionalization of PLA-NP with specific targeting ligands such as sialic acid and glycopeptides enhances their ability to cross the BBB [5, 26]. For instance, chitosan coating promote positive surface charge to PLA-NP, resulting in enhanced cell uptake [27]. In addition, PLA-NP exhibit biodegradable characteristics (unlike the metallic ones) [28]. A detailed evaluation of neurotoxicity mediated by different NP that are designed to be drug carriers in the CNS, is necessary and will contribute to the development of safer nanocarriers.

The current bioengineering revolution of cell culture makes organotypic cell models, which overcome many limitations of traditional cell culture, increasingly available [29]. Three-dimensional (3D) in vitro models are the most novel approach in this development, as they present closer cell-to-cell interactions, often include different cell types, and can better reproduce in vivo physiology [30–33]. 3D CNS cultures have demonstrated advantages compared to two-dimensional (2D) cultures, such as increased cell survival and differentiation and better reproduction of electrical activity [30, 34]. Moreover, NP delivery efficacy differs dramatically between 2D and 3D culture systems [35]. In addition, the use of 3D CNS models derived from human cells, especially human pluripotent stem cells, provide more human-relevant data compared to animal models, due to inter-species differences [36]. Although, animal models used for systemic toxicity, NP uptake, distribution in different organs, internalization and stability studies, provide useful and important information, the human physiology and kinetics should be taking in account. Thus, more advanced human relevant models such as organ-on-a-chip technology, where several organotypic human cultures are combined on a chip, are the future of drug delivery and nanotoxicity studies [37, 38]. Also, this will scale-up the testing, since traditional animal testing is time-consuming expensive and low-throughput.

Taking in account the advantages of 3D organotypic cultures, increasing number of studies have used variety of such models to study nanotoxicity and NP neurotoxicity [39–43]. Hoelting and collaborators developed an in vitro 3D neuronal model derived from human embryonic stem cell (hESC) and showed that polyethylene NPs (PE-NP) penetrated into the neurospheres and impacted gene expression at non-cytotoxic concentrations [42]. In the same way, Zeng and collaborators developed an in vitro 3D model of human neural progenitor cells and showed that different types of polyamidoamine dendrimers NPs can penetrate into neurospheres, affecting cell proliferation and migration through different pathways [43]. To the best of our knowledge, toxicity of gold and PLA-NP were not extensively studied in 3D brain models. However, they represent a potential group of NP that can be used for drug delivery in CNS, therefore, studying their neurotoxicity is essential [44, 45]. The toxicity of Au-NPs and PLA-NPs for CNS were previously investigated in vivo. Injection of Au-NP did not promote morphological modifications or death in mice retinal cells [46]. However, Au-NP treatment in rats increased the number of GFAP- positive astrocytes in hippocampus and cerebral cortex. In addition, an increasing diameter of cell body and length of foot process of astrocytes in animals challenged with Au-NP was observed [47]. PLA-NPs, polymeric NPs did not show in vivo toxicity and considered to be relatively safe. For example, Bejjani et al. have shown that these NPs did not alter rat retinal structure or induced cell death after intraocular injection [48]. However, it should be noted that PLA-NP were not investigated as extensively as Au-NP.

Both NPs are used for in vivo drug delivery. Previously, Au-NPs were used to IgG delivery in rabbit retina [49] and as adjuvant to improve immunization in spinal cord of injured rats [50]. PLA-NPs were used to carry Gag p24 HIV-1 antigen to increase immune response in mice [51] and T-cell responses on HIV-infected patients [52]. PEG-PLA-NPs were also used to delivery betamethasone in cochlea, promoting attenuation of cochlear hair cells loss induced by traumatic noise in mice [53].

Thus, in this study we focused on two types of NP: the metallic Au-NP already widely used for drug delivery and the biodegradable PLA-NP. We evaluated cellular effects of Au-NP functionalized with sodium citrate (Au-SC) and polyethylene glycol (Au-PEG), and PLA-NP, using two human 3D CNS in vitro models: (i) 3D LUHMES (Lund human mesencephalic) spheroids and (ii) human iPSC-derived brain spheroids (BrainSpheres). Both models have shown to be reproducible in content, size and shape; forming spheroids of approximately 250 and 350 μm, respectively [54]. LUHMES is an immortalized dopaminergic precursor cell line, derived from healthy human 8-week-old embryonic mesencephalic tissue, which rapidly differentiates into pure dopaminergic neurons [55, 56]. BrainSpheres derived from human iPSCs is a multicellular 3D brain model that contains various types of neurons, astrocytes, and oligodendrocytes, and show spontaneous electrical activity and myelination [54, 57]. By using these two models in parallel, we can compare a single-cell type 3D model with a more complex 3D system. After NP characterization, we evaluated NP uptake, morphological and molecular alterations such as viability and mitochondrial membrane potential, genes related to cytotoxicity and oxidative stress and secretion of cytokines, chemokines, and growth factors in both 3D models.

## Results

### NP characterization

**Fig. 1:**
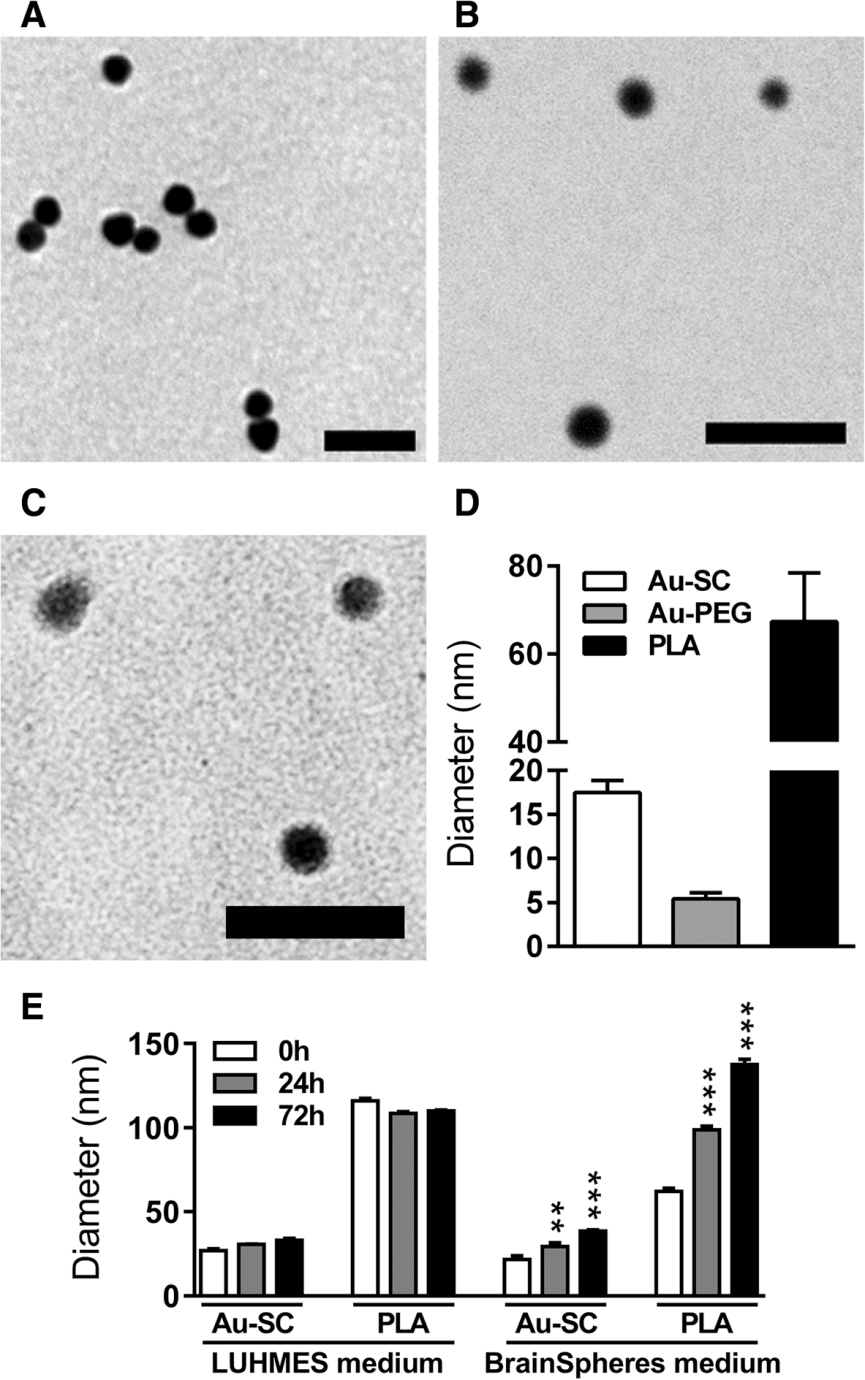
NP characterization. Representative images obtained by TEM of **a** Au-SC, **b** Au-PEG and **c** PLA-NP. **d** Quantification of NP size by TEM. **e** Z-average hydrodynamic diameter values of 6 μg/mL Au-SC and 20 μg/mL PLA-NP diluted in LUHMES and BrainSpheres culture media, analyzed by DLS after 0, 24 and 72 h of incubation. Results are expressed as mean (±SD). Each experimental group corresponds to the analysis of three independent experiments with three replicates. Statistical significance was analyzed by one-way ANOVA followed by Bonferroni’s multiple comparisons post-test (**p < 0.01, ***p < 0.001). Scale bar **a** 100 nm, **b** 25 nm and **c** 200 nm

In order to assess NP diameter, micrographs of Au-SC (Fig. 1a), Au-PEG (Fig. 1b) and PLA-NP (Fig. 1c) were acquired by TEM. Diameter of Au-SC, Au-PEG and PLA-NP was 17.5 ± 1.4, 5.4 ± 0.7 and 67 ± 3 nm, respectively (Fig. 1d). Hydrodynamic diameter of 6 μg/mL Au-SC and 20 μg/mL PLA-NP was assessed by DLS in medium without cells just after dilution and was 27 ± 1 and 116 ± 1.5 nm (0 h, 3D LUHMES medium) and 21 ± 2 and 62 ± 2 nm (0 h, BrainSpheres medium), respectively. Although, Au-SC and PLA-NP were larger in 3D LUHMES medium at 0 h, the diameters of these NP remained stable after 24 and 72 h. In contrast, Au-SC and PLA-NP diameters increased over time (Au-SC: 29 nm at 24 h to 38 nm at 72 h; PLA-NP: 99 nm at 24 h to 137 nm at 72 h) in BrainSpheres differentiation medium (Fig. 1e). Due to the small diameter of Au-PEG (~ 5 nm), they fall below the accurate range for DLS, as previously informed by the Au-PEG manufacturer, and therefore this measurement was not obtained for this NP type.

### 3D LUHMES and BrainSpheres production

**Fig. 2:**
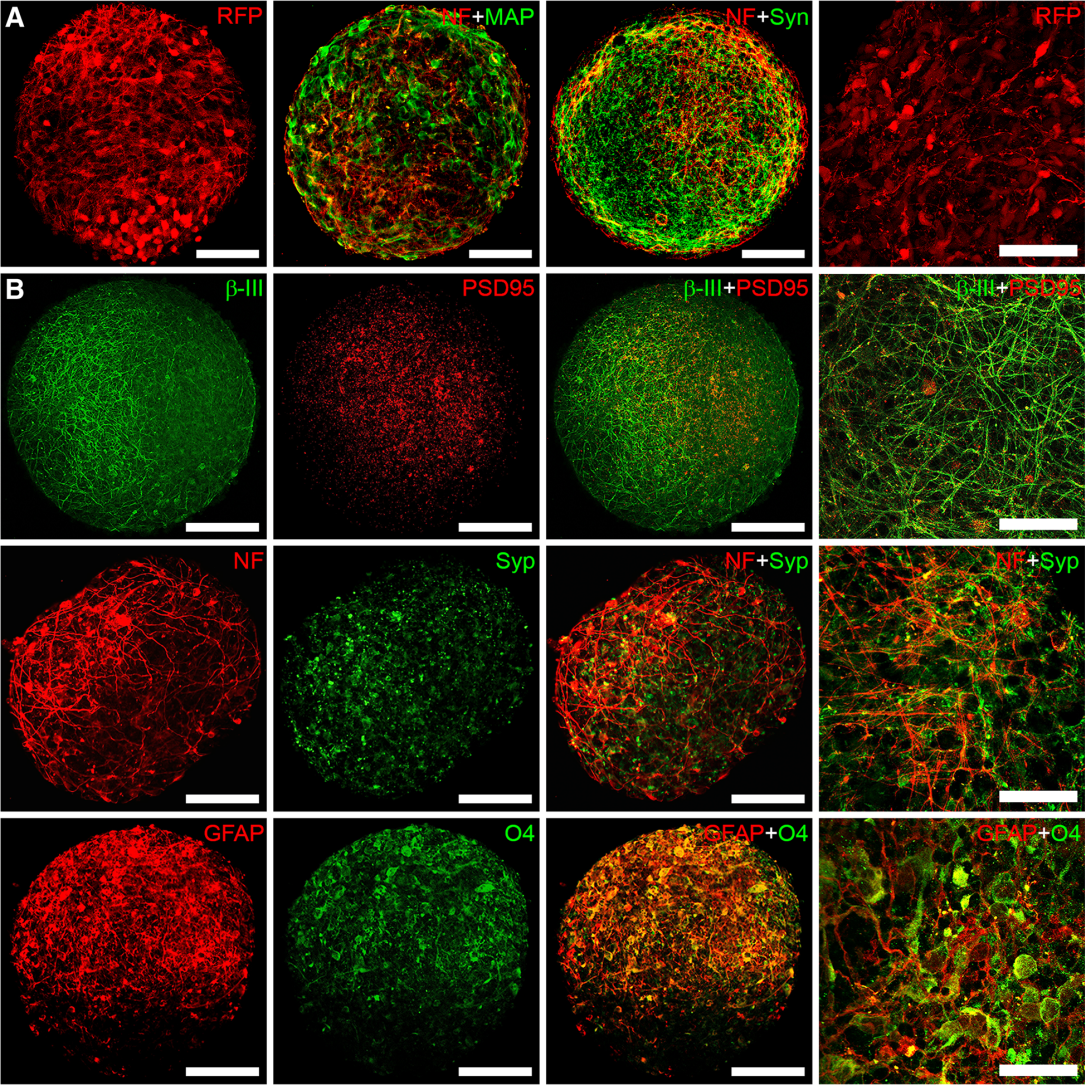
Confocal Images of **a** LUHMES after 7 days of differentiation, expressing RFP or stained with neuronal markers MAP2 (green), neurofilament (red), synaptophysin (green); and **b** 4-week BrainSpheres expressing different neural cell type markers: β-III- tubulin/PSD95, neurofilament/synaptophysin, GFAP/O4. Scale bars 100 μm for the first three panels and 50 μm for the last panel with higher magnification

3D LUHMES and BrainSpheres were produced by constant gyratory shaking as previously described [54, 56, 58–60]. Both LUHMES and BrainSpheres are highly reproducible in size (200–250 μm for LUHMES and 300–350 μm for BrainSpheres) and cellular composition from batch to batch and experiment to experiment. Due to their small size they do not develop a necrotic core as many bigger in size organotypic models do [54, 56, 58–60]. That makes these two models very suitable to use for neurotoxicological studies. At day 7 of differentiation, 3D LUHMES expressing RFP (red fluorescent protein) exhibited neuronal morphology with several cell projections and expressed markers of mature neurons, such as MAP2, neurofilament and synaptophysin (Fig. 2a). At 4 weeks of differentiation, BrainSpheres expressed different neural markers consistent with phenotypes of neurons (β-tubulin, neurofilament, synaptophysin, PSD95), astrocytes (GFAP) and oligodendrocytes (O4), as shown in Fig. 2b).

### NP uptake in 3D LUHMES and BrainSpheres

**Fig. 3:**
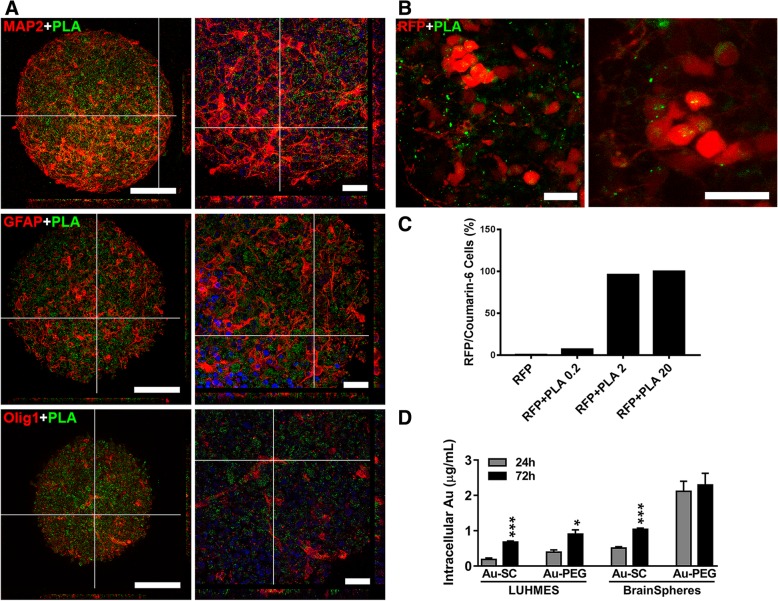
NP uptake by 3D LUHMES and BrainSpheres. Representative images acquired by confocal microscope after 72 h exposure to 20 μg/mL PLA-NP (green) of **a** BrainSpheres stained with MAP2, GFAP, OLIG-1 (red) and Hoechst33342 (blue nuclei), with the Scale bars 100 μm (left panels) and 25 μm (right panels); and **b** 3D RFP-LUHMES (red), PLA-NP (green), scale bar 20 μm. **c** Flow cytometry of RFP-LUHMES, treated with 0, 0.2, 2 and 20 μg/mL PLA-NP. Red and green fluorescence was quantified in FL2 and FL1 channels, respectively. The bars represent % of RFP/PLA double positive cells in each sample. **d** Intracellular levels of Au in 3D LUHMES and BrainSpheres after 24 and 72 h exposure to 6 μg/mL Au-SC and 20 μg/mL Au-PEG quantified by ICP-MS. Results are expressed as mean (±SEM). Each experimental group corresponds to the analysis of three independent experiments with three replicates. Student’s *t*-test was used to compare 24 and 72 h treatment groups (*p < 0.05, ***p < 0.001).

Since PLA-NP are conjugated to green fluorescent dye, coumarin-6, we analyzed their penetration into the spheroids by confocal imaging and flow cytometry. After 72 h exposure, uptake of 20 μg/mL PLA-NP by BrainSpheres (Fig. 3a) and 3D RFP-positive LUHMES (Fig. 3b) was visualized by confocal imaging. For better visualization, BrainSpheres were stained for neuronal (MAP2), astrocyte (GFAP) and oligodendrocyte (OLIG-1) markers and show even distribution of PLA-NP throughout spheroids reaching the core. The Additional files 1 and 2 show the internalization of PLA in RFP-positive LUHMES in more details (see Additional files 1 and 2).

In order to quantify the internalization and also demonstrate concentration-dependent internalization, we performed flow cytometry that allows to measure co-localization of red signal from RFP protein in LUHMES and green signal from PLA-NP. Thus, RFP-expressing 3D LUHMES were exposed to 0, 0.2, 2, and 20 μg/mL PLA-NP for 72 h, then spheroids were dissociated and fixed for subsequent analysis of co-localization between RFP (red) and PLA-NP (green) using flow cytometry. Results indicated increased co-localization between PLA-NP and RFP-expressing LUHMES cells in a concentration-dependent manner. Only 7.2% cells showed co-localization after exposure to 0.2 μg/mL while 96% cells co-localized with PLA-NP at 2 μg/mL, reaching 100% at 20 μg/mL (Fig. 3c and Additional file 3 Figure S1A). In addition, we assessed internalization with ImageStreamX Mark II imaging flow cytometer (Amnis), which allows not only to analyze the shift of the fluorescence intensities but also to obtain an image of every cell in the flow and analyze intracellular localization of NP. Trypan blue staining was included to quench autofluorescence and fluorescence of NPs, which might be just attached to the outer side of the membrane. Additional file 3: Figure S1B and C show representative images of unstained RFP-LUHMES and RFP- LUHMES treated with 20 μg/mL PLA NP for 24 h respectively. The punctuated staining inside the cells indicates internalization of PLA NP. Treatment with trypan blue did not shift the green fluorescence intensity significantly (compare median of RFP + PLA (14,014) vs. RFP + PLA + Trypan (11,989) suggesting that the green fluorescence signal is coming from the inside of the cells (Additional file 3: Figure S1D). Altogether, these data showed that NP penetrated throughout both models; although some NPs remained in the extracellular space, some PLA-NP were internalized by cells throughout the spheroids.

Au-SC and Au-PEG in both 3D models were assessed by ICP-MS, a method to study cellular uptake of metallic NP [61–64]. Our results showed Au-NP uptake in both models after 24 and 72 h exposure. Uptake of both Au-NP types was delayed in 3D LUHMES (increased after 72 h vs. 24 h exposure). In BrainSpheres, Au-SC uptake increased after 72 h compared to 24 h, while levels of internalization of Au-PEG at 24 and 72 h exposure time points were similar (Fig. 3d). Although, due to method limitations we cannot exclude the possibility of Au-NP absorption on the surface of the cells. The viability tests performed below indirectly confirm the presence of the Au-NP within the cells.

### Effects of NP on mitochondrial membrane potential (MMP) and cell viability in both 3D human neural models

**Fig. 4:**
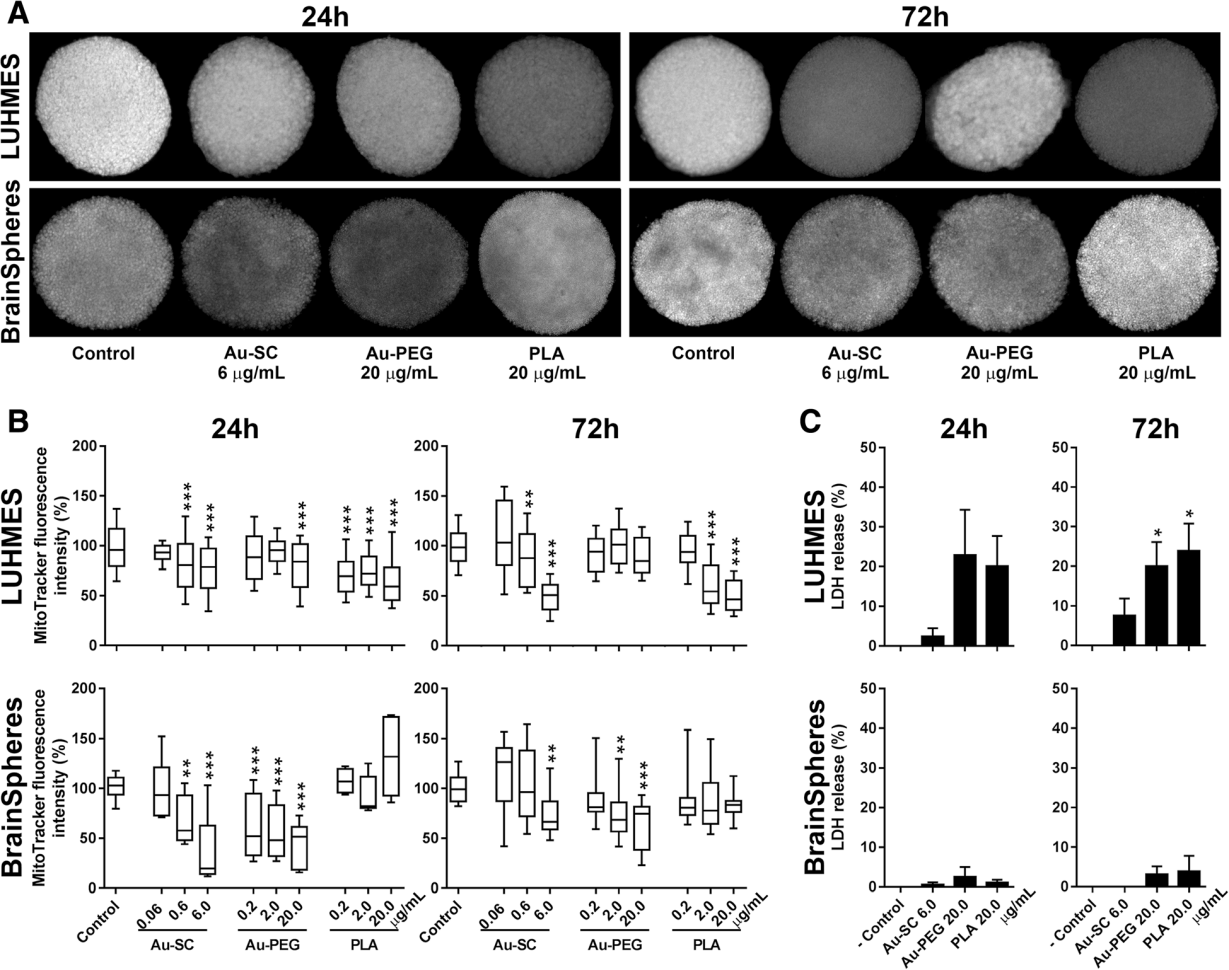
NP effect on MMP and cell viability in 3D human neural models. **a** Representative images of MitoTracker® staining in 3D LUHMES and BrainSpheres after 24 and 72 h exposure to Au-SC (6 μg/mL), Au-PEG (20 μg/mL) and PLA-NP (20 μg/mL). Reduction in the Mitotracker fluorescence intensity upon treatment with NP represents the reduction in uptake of the fluorescence probe due to impaired mitochondrial membrane potential. **b** MMP levels in 3D LUHMES and BrainSpheres after 24 and 72 h exposures to Au-SC (0.06, 0.6 and 6 μg/mL), Au-PEG (0.2, 2, 20 μg/mL) and PLA-NP (0.2, 2 and 20 μg/mL) normalized to the untreated spheroids controls. **c** Percent of LDH release in 3D LUHMES and BrainSpheres models after 24 and 72 h exposures to Au-SC (6 μg/mL), Au-PEG (20 μg/mL) and PLA-NP (20 μg/mL). ‘- Control’ corresponds to LDH released from untreated spheroids. Results are expressed as mean (±SEM). Each experimental group corresponds to three independent experiments imaging at least 20 spheroids. One-way ANOVA with Bonferroni’s multiple comparisons post-test was used for analysis of statistical significance (*p < 0.05, **p < 0.01, ***p < 0.001)

Both 3D LUHMES and BrainSpheres were exposed to PLA, Au-SC, and Au-PEG at different concentrations for 24 or 72 h, followed by analysis of MMP using MitoTracker® Red CMXRos (Fig. 4a, b). After 24 h exposure to 0.6 and 6 μg/mL Au-SC, MMP was reduced in both models in a concentration-dependent manner (Fig. 4b). After 72 h, a further decrease in MMP was observed in 3D LUHMES while BrainSpheres showed reduction only at 6 μg/mL. After 24 h exposure, only the highest concentration of Au-PEG (20 μg/mL) led to reduced MMP levels in 3D LUHMES, with no significant reduction at 72 h exposure. On the contrary, BrainSpheres showed a strong concentration-dependent reduction of MMP after 24 and 72 h exposure to Au-PEG. PLA-NP reduced MMP in 3D LUHMES at all concentrations after 24 h exposure, which reversed at 0.2 μg/mL and further decreased at 2 and 20 μg/mL after 72 h exposure. MMP of BrainSpheres was not affected by any of the PLA-NP tested concentrations. Taken together, we observed stronger acute than prolonged effects of tested NP on mitochondria functionality in BrainSpheres, while 3D LUHMES sensitivity to these NP was generally amplified with increased exposure concentrations and time. These data suggest different susceptibility of these 3D models to the studied nanomaterials, most probably due to the differences in cellular complexity of the models (Fig. 4a, b).

LDH release, a marker of cellular membrane damage and cell death, was measured at the highest concentrations of NP. The viability of 3D LUHMES was significantly affected (~ 25% cell death) by Au-PEG and PLA-NP after 72 h exposure. The studied NP did not affect BrainSpheres viability at the tested concentrations (Fig. 4c).

As expected, reduction in MMP was observed even without significant LDH release. Since mitochondria membrane depolarization occurs much earlier than cellular membrane lysis in the cascade of cell death events, it can be reversible [65–67], and, therefore, does not necessarily lead to cell death.

### Morphology changes in 3D LUHMES and BrainSpheres after NP treatment

**Fig. 5:**
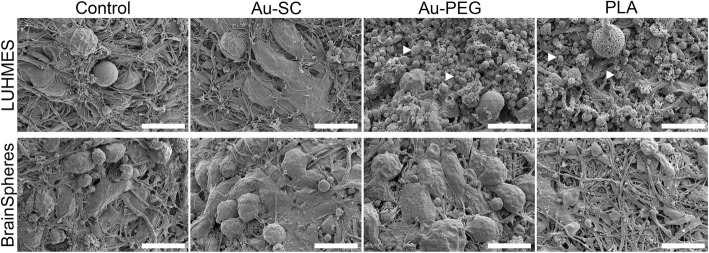
Morphology of 3D LUHMES and BrainSpheres exposed to Au-SC (6 μg/mL), Au-PEG (20 μg/mL) and PLA-NP (20 μg/mL) for 72 h. Control represents untreated spheroids. White arrowheads indicate cell debris. Scale bars 10 μm

The morphology of both spheroid models was analyzed by scanning electron microscopy (SEM) after 72 h treatment with the highest NP concentrations (Au-SC (6 μg/mL), Au-PEG (20 μg/mL) and PLA-NP (20 μg/mL)). Morphology of 3D LUHMES was affected by Au-PEG and PLA-NP, but not by Au-SC. Significant cell debris attached to the spheroids and less neuronal projections were observed (Fig. 5, depicted with arrowheads). BrainSpheres did not show any alterations under the investigated conditions, which correlates with above cytotoxicity data (Fig. 5).

### NP effect on expression of oxidative stress related genes in BrainSpheres

**Fig. 6:**
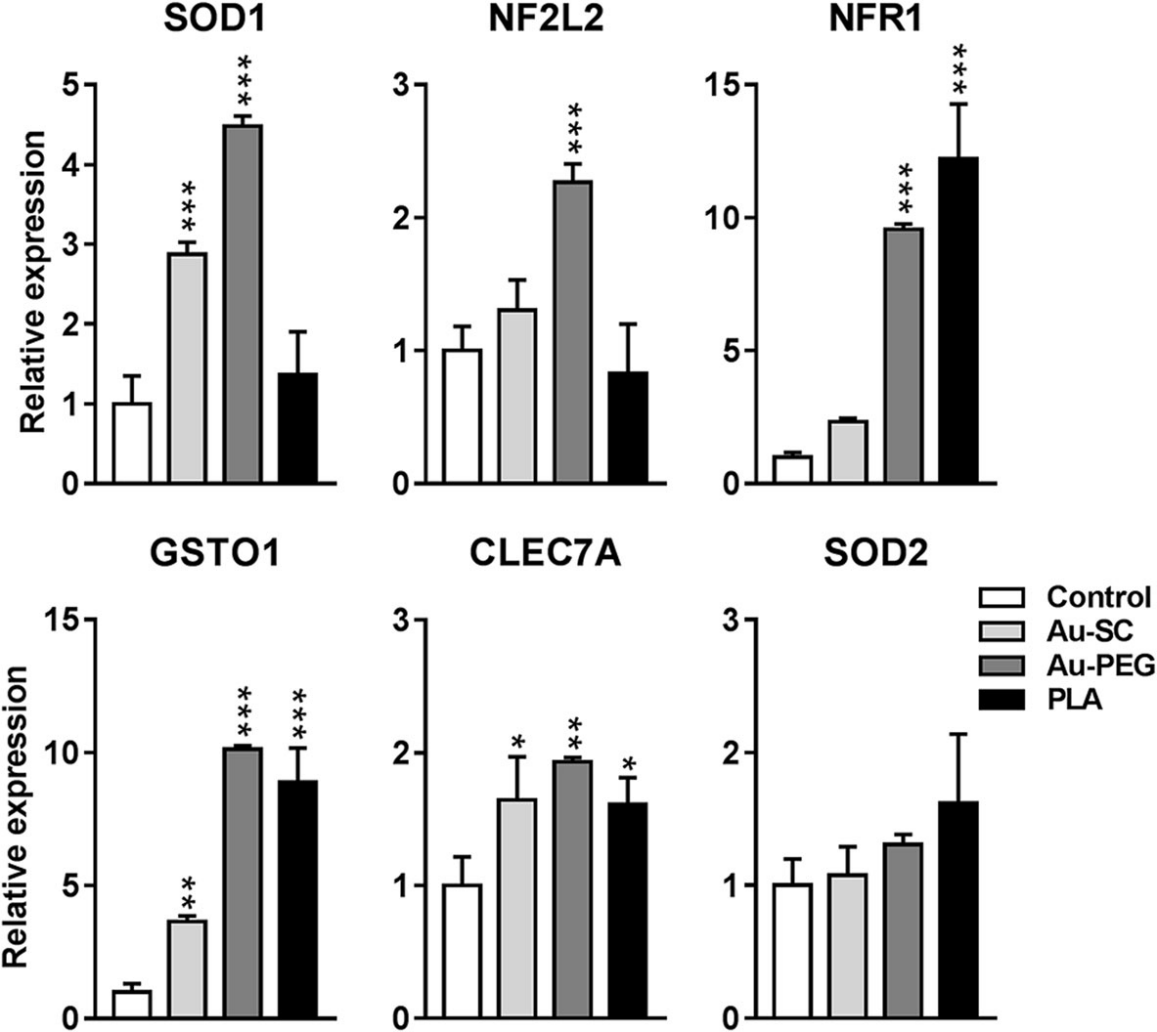
Effect of NP on expression of genes related to ROS regulation in BrainSpheres. Graphs showing the relative expression of *SOD1*, *SOD2*, *NF2L2*, *GSTO1*, *NFR1* and *CLEC7A* after exposure to Au-SC (6 μg/mL), Au-PEG (20 μg/mL) and PLA-NP (20 μg/mL) for 72 h normalized to the expression of the genes in the untreated control spheroids. Data was collected from three independent experiments and represents fold changes (FC ± SEM, *n* = 3). One-way ANOVA with Bonferroni’s multiple comparisons post-test was used to analyze the statistical significance (*p < 0.05, **p < 0.01, ***p < 0.001)

We further investigated changes in gene expression related to ROS regulation: *SOD1*, *SOD2*, *NF2L2*, *GSTO1,* and *NFR1* are involved in antioxidant responses and *CLEC7A* is associated with ROS production and inflammation. BrainSpheres and 3D LUHMES were exposed to Au-SC (6 μg/mL), Au-PEG (20 μg/mL), and PLA-NP (20 μg/mL) for 72 h, then gene expression was analyzed by Real-Time qPCR. In BrainSpheres, *SOD1* expression was increased by Au-SC and Au-PEG, but not PLA, whereas *NF2L2* was increased only by Au-PEG. *NFR1* expression was up-regulated by Au-PEG and PLA-NP, while expressions of *GSTO1* and *CLEC7A* were up-regulated by all NP. *SOD2* expression was not altered upon NP challenge (Fig. 6). These data showed that the exposure of BrainSpheres to NP increased expression of genes related to oxidative stress protection in BrainSpheres, and the strongest effect was observed with Au-PEG, in line with the higher uptake of this NP. The perturbations in 3D LUHMES were less dominant. No significant effects on the expression of *SOD1*, *SOD2* and *GSTO1* were observed; *CLEC7A* was undetermined. *NF2L2* was slightly elevated by AU-PEG, while *NFR1* expression was downregulated by AU-SC and Au-PEG NP (Additional file 4: Figure S2).

### NP influence on release of chemokines, cytokines and growth factors in 3D human neural models

**Fig. 7:**
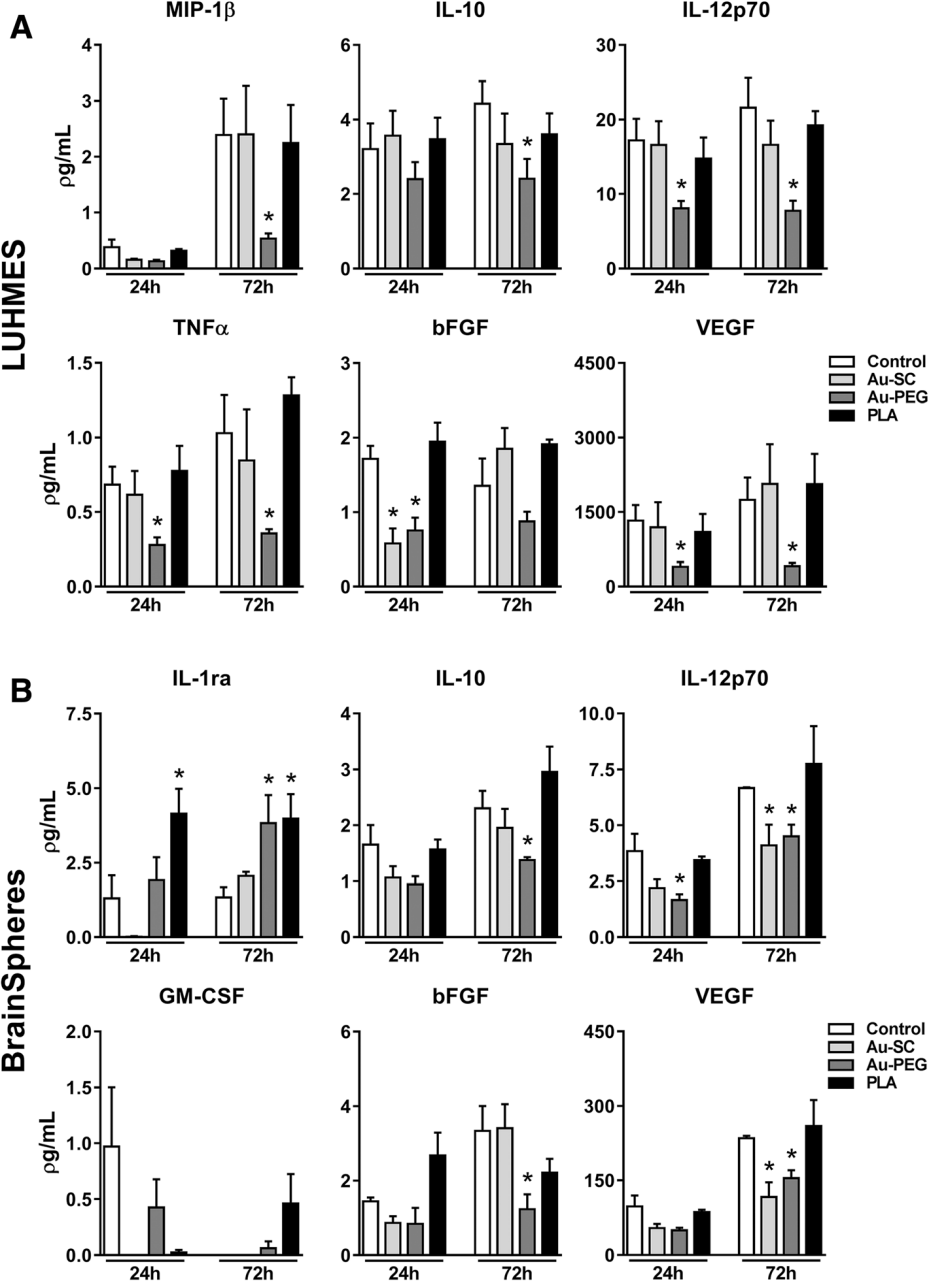
NP influence release of chemokines, cytokines and growth factors in 3D human neural models. Graphs showing the levels of different secreted mediators after exposure to Au-SC (6 μg/mL), Au-PEG (20 μg/mL) and PLA-NP (20 μg/mL) for 24 and 72 h in comparison to untreated control spheroids. **a** 3D LUHMES (MIP-1β, IL-10, IL-12p70, TNFα, bFGF and VEGF) and **b** BrainSpheres (IL-1ra, IL-10, IL-12p70, GM-CSF, bFGF and VEGF). Data were collected from three independent experiments with three technical replicates and represents mean (± SEM). One-way ANOVA with Bonferroni’s multiple comparisons post-test was used to analyze the statistical significance (*p < 0.05)

Analysis of multiple secreted products from both 3D models exposed to 6 μg/mL Au-SC, 20 μg/mL Au-PEG or 20 μg/mL PLA-NP for 24 and 72 h showed alterations in the levels of some mediators (Fig. 7). In general, neural cells produce lower levels of chemokines and cytokines compared to cells of the immune system, but such levels are critical to maintaining their homeostasis and, consequently, the microenvironment. Thus, any imbalance may affect their physiological behavior [68].

From all conditions tested, Au-PEG had the strongest effect on cytokines release in 3D LUHMES cultures. It significantly downregulated the levels of all cytokines tested. No significant changes in cytokine release were observed in LUHMES cultures treated with Au-SC and PLA-NP (Fig. 7a). TGF-β1, TGF-β2, and TGF-β3 levels were not significantly altered in 3D LUHMES (Additional file 5: Figure S3).

**Fig. 8:**
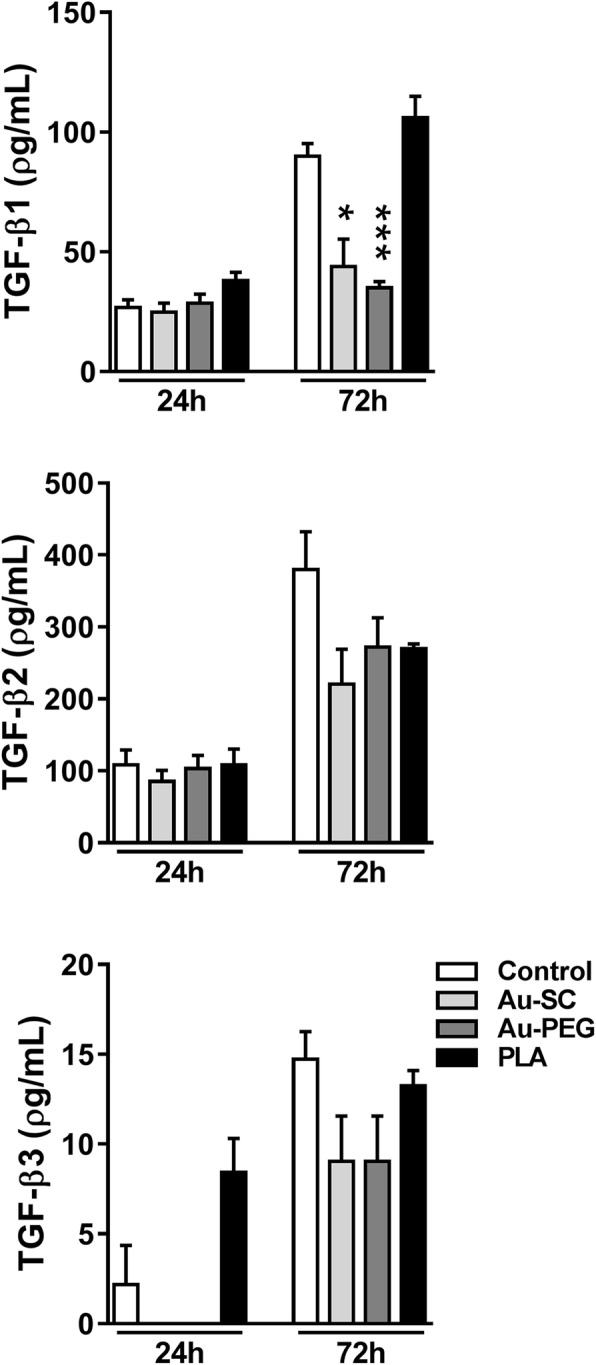
Influence of NP on release of TGF-β isoforms in BrainSpheres. Graphs show the levels of secreted TGF-β1, TGF-β2 and TGF-β3 after exposure to Au-SC (6 μg/mL), Au-PEG (20 μg/mL) and PLA-NP (20 μg/mL) for 24 and 72 h in comparison to untreated control spheroids. Each experimental group corresponds to the analysis of three independent experiments with three replicates and represents mean (± SEM). One-way ANOVA with Bonferroni’s multiple comparisons post-test was used to analyze the statistical significance (*p < 0.05, ***p < 0.001)

BrainSpheres were not as sensitive to Au-PEG with only two cytokines (IL-10, IL12p70) and two growth factors (bFGF, VEGF) downregulated in these cultures, while IL-1ra was upregulated by Au-PEG and PLA-NP. In addition, Au-SC reduced the levels of IL12p70 and VEGF in BrainSpheres (Fig. 7b). In BrainSpheres, TGF-β1 levels were reduced by Au-SC and Au-PEG after 72 h. These NP also eliminated TGF-β3 in the first 24 h, but not after 72 h. PLA-NP did not affect the levels of these soluble mediators (Fig. 8). Altogether, these data suggested different responses in the profile of chemokines, cytokines, and growth factors to the studied nanomaterials in both models, which are both devoid of immune cells. The very low levels and frequent reductions in cytokine levels compared to control are difficult to interpret. However, there seems to some correspondence of cytokine patterns in response to the NP between the two models, which corroborates the findings. At this stage, these findings are descriptive and mainly illustrate that cytokines could be highly sensitive quantitative biomarkers for perturbation of cells by NP. Further work is needed to understand the mechanism and relevance of these changes.

## Discussion

The use of NP as carriers promises advantages such as better drug stability, bioavailability, improved dosing and reduced side effects. Many NP drug delivery systems are being developed, such as Au-NP conjugated to siRNA against NADPH Oxidase 4, Au-NP carrying plasmid DNA encoding for murine IL-2, PLA-NP carrying Gag p24 HIV-1 antigen, B6 peptide conjugated to PEG-PLA-NP encapsulating the neuroprotective peptide NAPVSIPQ to Alzheimer’s disease mice model, and others [51, 69–71]. However, nanotoxicity must be controlled before administration [3, 72]. From the variety of NP used for drug delivery, Au-NP are one of the most widely used [73]. However, recent studies have shown their potential to induce (neuronal) cytotoxicity and neuroinflammation [23, 24]. PLA-NP emerged as an alternative for CNS drug delivery due to their biocompatibility, biodegradability, and drug release kinetics [74]. Therefore, in this study we decided to use Au-NP as a reference nanomaterial, since they are well established and described delivery system, and compare toxicity of Au-NP with an alternative system – PLA-NP.

NP derived from different materials, including Au-NP and PLA-NP are able to cross the BBB, especially if modified with specific ligands, increasing the penetration efficiency. They reach the CNS what makes them excellent potential drug carriers for reaching the cerebral parenchyma [3, 5–8]. However, reported Au-NP toxicity to the CNS includes apoptosis and alterations in retinal layers structure, microglia activation, increase of neuronal excitability in CA1 region of hippocampus and the potential to aggravate seizure activity [75–77]. The use of PLA-NP for drug delivery is a relatively recent approach, showing positive results in treating hearing loss and Alzheimer’s disease in animal models, without inducing toxicity [53, 71]. Functionalization of NPs is important to ensure their colloidal stability and biocompatibility. It should be kept in mind, that these surface modifications may affect cellular toxicity. For instance, cationic functionalization of Au-NPs is more toxic than the neutral and anionic ones, and toxicity will depend on the charge magnitude/intensity [78, 79]. Another study showed that citrate and biotin modified Au-NPs were non-toxic up to higher concentrations, whereas CTAB functionalization was toxic [80]. NP used in this study were not functionalized with cytotoxicity inducing cationic ligands. No cytotoxicity was reported for PEG functionalization [81, 82].

Several 3D models have been developed and used to study NP toxicity, drug discovery and delivery [31, 33, 35, 37–40]. Human-derived 3D neural in vitro models, which mimic complex human CNS interactions are a promising tool [54, 83–85] that can be applied to study the potential cytotoxic effects of nanocarriers. The advantages of the two models presented here over the other existing in vitro organotypic brain models lie in the high standardization of the spheroid generation procedures: the spheres generated with gyratory shaking are very homogeneous in size, form and cellular composition as was shown in our previous publications [54, 56, 58, 59]. Although, other brain organotypic models are more complex in representation of the organ morphology [84, 85], we have chosen the models described here because the homogeneity is a key feature in studying toxicity. In the future, administration of NP in the other brain organoids may confirm the findings presented here.

In this study we used Au-SC NP in concentrations up to 6 μg/mL, Au-PEG and PLA up to 20 μg/mL. The direct comparison of NP brain concentrations in vivo with in vitro nominal and final concentrations is challenging as well as quantification of final NP concentration in the target organ. However, there are studies that quantified the amounts of Au-NPs in the mice brain using ICP-MS, the gold standard method for metallic detection in cells and organs. Khlebtsov and Dykman, 2011 adapted the results from [86] showing that after oral administration of 200 μg/mL Au-NPs with 4, 10 and 28 nm in size, these NPs were detected and quantified in the brain: from 10 ng/mL (4 nm Au-NPs) to 1 ng/mL (28 nm Au-NPs) [87]. Comparing to our data, we used 4 nm Au-NPs in vitro and detected up to 1000 ng in LUHMES and up to 2000 ng in BrainSpheres per well (with around 50 LUHMES spheroids with 200–250 μm in diameter and around 80 to 100 BrainSpheres with 300–350 μm in diameter per well). (Fig. 3d). These NPs were detected in lung, heart, kidney, spleen, liver, small intestine and stomach in different concentrations [87]. It should be kept in mind that the 200 μg/mL Au-NPs in the aforementioned study were administrated via water bottles for 7 days, without control of daily ingestion. It is possible that Au-NPs may have precipitated and deposited on the bottom of the bottle that may result in lower concentrations of NP in target organs. Jain et al., have shown that rats treated intravenously with 60 μg/mL of PLA-NP had particles in the brain [88] besides accumulation in the liver, kidney, lungs, heart and spleen. However, since PLA NPs are not metallic and may degrade quickly, they cannot be detected by ICP-MS, that makes it difficult to measure their concentrations in the organs.

To characterize the interaction between NP, media and cells in vitro, we assessed NP size, penetration throughout the spheroids and internalization. Our NP characterization showed that Au-SC and PLA-NP did not change in size over time in LUHMES differentiation medium. Although, immediately after resuspension of NP in LUHMES differentiation medium, NP size was higher than previously measured by TEM in water, they were then stable in size for 24 and 72 h (Fig. 1c). However, in BrainSpheres differentiation medium, we observed a gradual increase in NP size for both Au-SC and PLA-NP. In both media, the immediate (3D LUHMES) or delayed (BrainSpheres) increase in NP size can be attributed to aggregation or the formation of a protein corona due to NP interaction with proteins in cell culture medium. It is possible that proteins from the medium supplements attach to the NP, affecting their hydrodynamic analysis. More work is needed within the characterization field to better predict how these media alter NP physico-chemical properties. Due to the proprietary composition of cell culture supplements, further analysis to identify these proteins was not possible. However, a protein corona could facilitate cellular NP uptake, as is known to occur in blood circulation [89]. Studying the composition of the protein corona would be necessary to assess and improve drug delivery for CNS therapies. A recent study showed that artificial apolipoprotein E4 adsorption to NP forming corona increases NP translocation through BBB, improving the brain parenchyma accumulation threefold when compared to undecorated particles [90].

A major question for the use of 3D organotypic cultures to assess NP toxicity is, whether the NP can actually penetrate the spheroid and reach the inner cell mass. The 3D models, studied here, were, indeed, able to take up the three NP types. As quantified by flow cytometry, PLA-NP were internalized by LUHMES in a concentration-dependent manner, with 2 μg/mL being sufficient to penetrate 96% of cells. These results support the confocal images that showed PLA-NP in the core of both 3D models. Au-SC uptake was similar in both 3D models with increased levels after 72 h. Au-PEG showed the same result as Au-SC in 3D LUHMES, but the uptake in BrainSpheres was 5-fold higher after 24 h and stabilized after 72 h. The differences in Au-PEG internalization between the 3D models could be attributed to the presence of glial cells in BrainSpheres that may take up these NP more efficiently than neurons and/or regulate neuronal uptake as previously shown [91]. In fact, PEG functionalization contributes to a better NP uptake by neural cells, and their smaller diameter may be the reason for higher intracellular levels compared to Au-SC in BrainSpheres. This is in line with studies showing that PEGylation increases NP accumulation in the brain compared to non-PEG-coated NP [3, 92]. Therefore, PEG-coating and the presence of glial cells facilitating uptake could explain the high Au-PEG accumulation in BrainSpheres in the first 24 h and their maintenance until 72 h.

Due to the efficient NP internalization by neural cells in the 3D models, their potential to induce nanotoxicity needs to be considered. In 3D LUHMES, Au-SC but not Au-PEG significantly reduced mitochondria function (MMP). Au-PEG had only slight acute effects at the highest concentration tested, which was no longer significant after 72 h exposure. However, Au-PEG increased LDH release, possibly due to the high contact surface area of this NP that may contribute to neuronal lysis. In BrainSpheres, MMP was significantly reduced by both AuNP but no increase in LDH release was observed. In this model, glial cells may be involved in the AuNP clearance, reducing their availability and damage to neuronal cells.

PLA-NP reduced MMP and increased LDH release in 3D LUHMES, but showed no effect in BrainSpheres. The 3D LUHMES model is a monoculture model, consisting only of dopaminergic neurons, and may display higher susceptibility to harmful agents that induce mitochondrial damage, and cytotoxicity [93]. In fact, NP cytotoxicity has shown to be lower when neurons and astrocytes are co-cultured, compared to monocultures [94]. The absence of neuroprotective glial cells, together with the known susceptibility of dopaminergic neurons to mitochondria depletion, could contribute to the toxicity observed in this model from all studied NP. On the other hand, astrocytes in BrainSpheres as well as polymeric composition of PLA-NP may be critical to increase NP biocompatibility and cell tolerance. It is important to note that mouse prenatal exposure to NPs induces neurotoxicity during development and astrocytes reactivity. Maternal inhalation of carbon black increased GFAP levels in astrocytes from cortex and hippocampus offspring [95]. Indeed, exposition of mice from postnatal day 4–7 and 10–13, which is equivalent to human third trimester, to ultrafine particle air pollution promoted inflammation, neurotoxicity, gliosis and behavioral dysfunction [96]. In the line with that, we observed an increase in glia marker - S100β - after exposure of BrainSpheres to Au-PEG for 72 h (data not shown), suggesting an induction of gliosis by Au-PEG NP in BrainSpheres.

Mitochondrial dysfunctions are associated with ROS production, promoting cell stress and death [97]. NP can mediate this deregulation in neural cells [98]. Since AuNP decreased MMP without cell viability loss in BrainSpheres, we investigated the expression of genes involved in antioxidant responses and ROS production. Although all studied NP led to increased *CLEC7A* expression, related to ROS production and inflammation, *SOD1, NF2L2, GSTO1,* and *NFR1,* related to antioxidant responses in general, were increased by Au-PEG and PLA-NP but not by Au-SC. This activation of antioxidant genes may indicate that the cell activated antioxidant response as a reaction to the NP challenge in BrainSpheres. Different sources such as free radicals on NP surface and redox group from NP functionalization may cause the induction of ROS production and oxidative stress, depending on NP type. The differences of oxidative stress induction may activate different intracellular signaling pathways such as the activation of transcription factors and cytokines production and release [99].

These results showed that the studied NP do not necessarily induce toxicity directly but are able to unbalance cell physiology. Amongst these NP, Au-PEG had the strongest effect, in line with their increased uptake.

In BrainSpheres, exposure to NP did not decrease viability or lead to any apparent morphological alterations. However, AuNP affected mitochondrial activity and increased antioxidant genes with a possible activation of cell survival programs, which may be sufficient to maintain cell viability and morphology. In contrast, 3D LUHMES exposed to Au-PEG and PLA-NP showed morphological alterations on the surface of spheroids, which was confirmed by cell death measured by LDH release. These results reinforce the impact of glial cells in NP tolerance to toxic insults.

Glial and neuronal cells produce and secrete factors to maintain survival. Some of them are considered pro-inflammatory, but also important at physiological levels for neuron-glia communication [100]. The level of these factors can be altered in response to harmful agents. Au-PEG reduced most detected cytokines, chemokines, and growth factors in both 3D models. In 3D LUHMES, a reduction in MIP-1β, IL-10, IL-12p70, TNFα, bFGF and VEGF levels are in line with the observed effects on viability. In BrainSpheres, Au-PEG reduced the levels of IL-10, IL-12p70, bFGF, VEGF, and TGF-β1, as well as increased the IL-1ra levels. Au-SC affected fewer secreted mediators, probably due to their smaller contact area and low cell penetration rate compared to PEGylated AuNP. In CNS, IL-12 is manly related to pathogenesis of autoimmune diseases; VEGF to neurogenesis, neuronal migration, neuroprotection, and blood vessel growth; and TGF-β1 displays neuroprotective role and promotes glial scar and fibrosis, induced by acute and chronic brain injury [101–103]. IL-10 is an important anti-inflammatory mediator. The reduced levels of such mediators suggest that AuNP in a first interaction with neural cells may turn them susceptible to other subsequent harmful agents in the CNS. However, such alterations together with reduced MMP were not sufficient to induce cell death in BrainSpheres, possibly due to the increased expression of antioxidant genes and the neuroprotective role of astrocytes that may maintain cell survival.

PLA-NP had minimal effects on secreted cytokines, chemokines, and growth factors, restricted to IL-1ra increase in BrainSpheres. IL-1ra binds to IL-1 receptor in cell membranes, preventing IL-1 downstream signaling and activation of inflammation [104]. This change, however, was small and may not be relevant for the cell functionality. Further experiments will be conducted to better elucidate the cytokine findings described here.

Altogether, we consider PLA-NP a stealth nanomaterial in multicellular 3D human neural models, containing neuron and glial cells, without altering cell physiology and functionality and suitable for carrying drugs of interest in the CNS. In the future, we will address the cell-specific responses to the NP within the BrainSpheres such as reaction of glia cells (e.g., gliosis after exposure to AU-PEG, we observed in our preliminary data). In addition, we will make use of our recently developed method of introducing microglia into the BrainSpheres [58]. This will allow us to look into the inflammatory response such as cytokine release by immune cells in more details.

## Conclusions

**Fig. 9:**
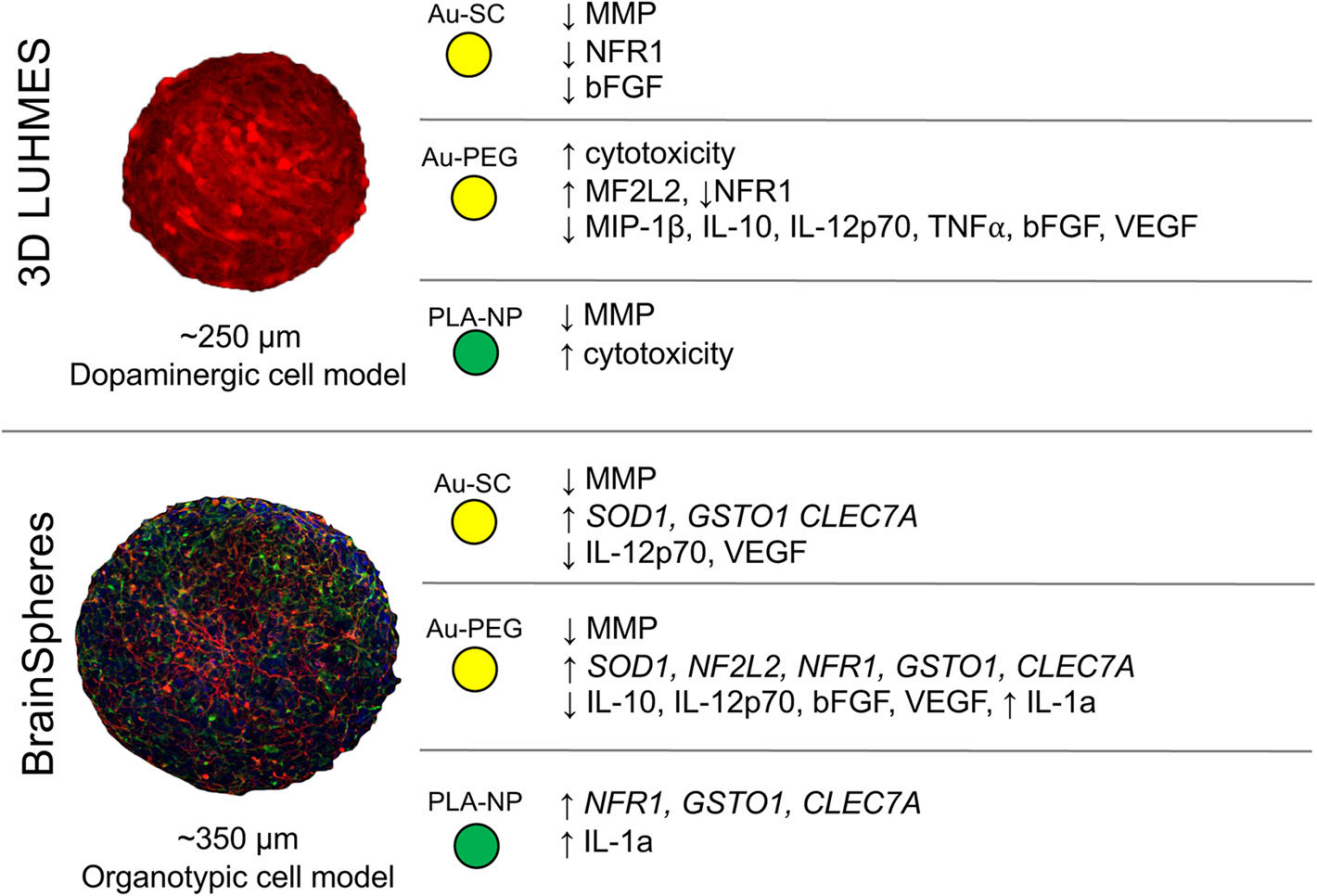
Summary of the observed results in 3D LUHMES and 3D BrainSphere models exposed to the NP

This work showed that 3D brain spheroid models are well suited to comparatively characterize NP neurotoxicity. The simpler, single-cell model was more sensitive to the toxic effects, in line with the lack of glia support to neurons. The use of multiple models, which encompass simplicity and physiological relevance, serves as tool for more NP drug-delivery focused research (Fig. 9).

In conclusion, we have demonstrated that AuNP, the most used nanocarrier, as well as PLA-NP, may be harmful to pure dopaminergic neurons at the highest concentrations tested, which might represent a risk to contribute to Parkinson’s disease development. In particular, in midbrain substantia nigra, where there is a high number of dopaminergic neurons, the use of nanocarriers should be carefully evaluated. However, this risk might be overestimated as the model lacks neuroprotection provided by astrocytes. Thus, BrainSpheres, are more appropriate model to study general neurotoxicity because they contain the glia, which can provide such neuronal support.

In a mixed population of neural cells within the BrainSpheres, the studied nanocarriers did not affect viability, likely due to the presence of glial cells and their participation in brain clearance. Our findings showed that AuNP promoted alterations in cell physiology that may contribute to increased susceptibility to other subsequent harmful agents in the CNS. Therefore, the use of AuNP as drug carrier in the CNS must be further evaluated. PLA-NP induced minor alterations in BrainSphere neural cell physiology, emerging as a safer alternative for brain drug delivery.

## Methods

### Nanoparticles and chemicals

Spherical monodisperse NP diluted in ultrapure water were used in this study. Au-SC were produced with 15 nm nominal diameter through reduction of 1% tetrachloroauric acid (Sigma Chem. Co.) by 1% sodium citrate aqueous solution (Merck KGaA), based on the Turkevich method revised by Kimling [105]. An Au-SC suspension was produced at 58 μg/mL and 1.7 × 10^12^ particles/mL concentration. Au-PEG 5-kDa at 1 mg/mL Au mass concentration (10^15^ particles/mL) were purchased from Nanocomposix (batch JMW1410). Green-fluorescent Coumarin-6 PLA-NP at 6.3 mg/mL polymer concentration (1.05 × 10^13^ particles/mL) were acquired from IBCP (Lyon, France). All NP were stored and protected from light. Dimethyl sulfoxide (DMSO), paraformaldehyde (PFA) and cell lysis buffer (CelLytic M) were from Sigma Aldrich.

### Nanoparticle characterization

NP were sonicated for 5 min and particle suspensions (10 μL of 1 μg/mL) were deposited on copper grids, air-dried and imaged in Tecnai G2 Spirit BioTwin 12 (Au-SC and Au-PEG) or LEO 912 Omega (PLA-NP) (FEI) transmission electron microscopes (TEM), both operated at 120 kV. NP diameter size were determined by ImageJ software (https://imagej.nih.gov/ij/index.html NIH). For analysis of NP hydrodynamic diameter, Au-SC (6 μg/mL), Au-PEG and PLA-NP (20 μg/mL each NP) were diluted in 3D LUHMES and BrainSpheres media and incubated in cell culture flasks without cells following the same conditions as for cell cultures. Then, 1 mL of sample was transferred to an appropriate cuvette for subsequent analysis of dynamic light scattering (DLS) in the Malvern Zetasizer Nano ZS apparatus (Malvern Instruments Ltd).

### Cell culture

#### 3D LUHMES

Wild-type and red fluorescent protein (RFP) genetically modified LUHMES human neuronal precursor cells [106] were kindly provided by Prof. Marcel Leist (University of Konstanz) and maintained and cultured as previously described [55, 56, 107]. Briefly, flasks were pre-coated with 50 μg/mL poly-L-ornithine and 1 μg/mL fibronectin (both from Sigma Aldrich) for 12 h. Cells were maintained in Advanced DMEM/F12 (ThermoFisher) supplemented with 2 mM L-glutamine (Sigma Aldrich), 1x N2 (ThermoFisher) and 40 ng/mL recombinant basic Fibroblast Growth Factor (bFGF, R&D Systems) (LUHMES proliferation medium), and passaged every 2–3 days. For 3D neuronal differentiation, cells were seeded in 6-well plates at 5 × 10^5^ cells/well in 2 mL Advanced DMEM/F12 supplemented with 2 mM L-glutamine, 1x N2, 1 mM dibutyryl cAMP (Santa Cruz), 2 μg/mL tetracycline (Sigma Aldrich) and 2 ng/mL recombinant human Glial cell line-Derived Neurotrophic Factor (GDNF, R&D Systems) (LUHMES differentiation medium). The spheroids were placed on an orbital shaker (ES-X, Kuhner shaker) with 50 mm orbit diameter at 80 rpm in a humidified incubator at 37 °C and 10% CO_2_. As per the differentiation protocol [107], paclitaxel (Sigma Aldrich) was added on day 3 to block proliferation and washed-out on day 5. On day 7, spheroid size was quantified using SPOT software 5.0 (Diagnostic Instruments Inc). 3D LUHMES were differentiated up to 10 days.

#### BrainSpheres

Neural progenitor cells (NPC) were differentiated from iPSC [108] and kindly provided by Professor Hongjun Song’s lab within our joint project [54]. iPSC were derived from C1 (CRL-2097) fibroblasts purchased from ATCC [108]. NPC were maintained in KO DMEM/F12 medium supplemented with 1x StemPro supplement (ThermoFisher), 20 ng/mL human bFGF (ThermoFisher), 20 ng/mL Epidermal Growth Factor (EGF, ThermoFisher), 4 mM L-Glutamine (ThermoFisher), 500 Units Penicillin and 500 μg Streptomycin (ThermoFisher). Half of the medium was replaced every 24 h. For BrainSpheres differentiation (previously described [54]), cells were mechanically detached when reached 100% confluence and seeded in 6-well plates at 2 × 10^6^ cells in 2 mL Neurobasal Electro medium (ThermoFisher) supplemented with B-27-electro (ThermoFisher), 10 ng/mL Brain-Derived Neurotrophic Factor (BDNF) and 10 ng/mL GDNF (Gemini), 4 mM L-glutamine (ThermoFisher), 500 Units Penicillin, and 500 μg Streptomycin (ThermoFisher). Cells were placed on an orbital shaker with 19 mm orbit diameter at 88 rpm into humidified incubator at 37 °C and 5% CO_2_. Medium was replaced every 48 h. After 4 weeks of differentiation, the BrainSpheres were used for the experiments. Spheroid size was quantified using SPOT software 5.0 (Diagnostic Instruments, Inc.).

### NP treatment

NP stock suspensions were diluted in differentiation media on the day of treatment to prepare following final concentrations: 0.06, 0.6 and 6 μg/mL of Au-SC; 0.2, 2 and 20 μg/mL of Au-PEG and PLA nanoparticles. The used Au mass concentrations were in the range of previous in vitro studies [75–77]. 3D LUHMES were treated with NP on day 7 of differentiation for 24 or 72 h. BrainSpheres were treated with NP after 4 weeks of differentiation for 24 or 72 h. Then, spheroid and supernatant samples were collected for endpoint measurements.

### Immunocytochemistry and confocal microscopy

3D cultures were fixed with 4% paraformaldehyde (PFA) for 1 h, washed 3 times with PBS and incubated for 2 h with blocking buffer (1% BSA, 5% goat serum, 0.15% saponin (Sigma Aldrich)). Samples were incubated 48 h with primary antibodies (1:200 mouse anti-MAP2 (Sigma Aldrich); 1:200 rabbit anti-GFAP (Dako) and 1:200 mouse anti-Olig1 (Millipore), 1:200 mouse anti-Synaptophysin (Sigma), 1:200 rabbit anti-PSD95 (Life Technologies), 1:1500 β-III-tubulin (Sigma Aldrich), 1:200 anti O4 (R&D Systems) diluted in blocking buffer) at 4 °C, followed by three washing steps and incubation with secondary antibodies (1:500 goat anti-mouse Alexa fluor 488 or 1:500 goat anti-rabbit Alexa fluor 568, diluted in blocking buffer, Molecular Probes) overnight. Then, samples were washed and incubated with Hoechst 33342 (1:10,000, Molecular Probes) for at least 1 h at room temperature (RT). After three washing steps, the samples were mounted on glass slides with Prolong Gold-antifade reagent (Molecular Probes) for confocal microscopy. Z-stacks started at the top of the sample were taking. Images were obtained with Zeiss LSM-510 (Zeiss) and Leica SP5 (Leica) confocal microscopes with identical time exposure and image settings.

### Flow cytometry

3D RFP-expressing LUHMES treated with different concentrations of PLA-NP for 24 h or 72 h were trypsinized with TryplE Express containing 4 units/mL DNase at 37 °C for 30 min on the shaker. 40 μg/mL Trypan Blue was added to subset of the samples to quench NP fluorescence from outside the cells. Then, samples were homogenized using a 1 mL syringe with a 26G3/8 needle. Cells were washed with PBS twice, fixed with 4% PFA for 30 min and the co-localization between 3D RPF-expressing LUHMES cells and PLA-NP green fluorescence was quantified using a FACSCalibur flow cytometer (BD) or with ImageStreamX Mark II imaging flow cytometer (Amnis) (in this case cells were imaged live). The instrument was calibrated using fluorescent beads and wild-type LUHMES cells were used as negative control to set the gates.

### Inductively coupled plasma mass spectrometry (ICP-MS)

AuNP stock solution was used to prepare the calibration solutions through serial aqueous dilutions for ICP-MS determination (NexION 300D, PerkinElmer). 1% (v/v) nitric acid (Merck) and 10 μg/L rhodium from Perkin-Elmer was added to the calibration solution, blank, and samples to improve analytical performance. The Au^197^ isotope was measured. For quantification of intracellular Au mass, spheroids of both models were treated for 24 or 72 h with Au-SC or Au-PEG. Then, spheroids were collected and lysed (CelLytic M lysis buffer, Molecular Probes) for analysis.

### Mitochondrial membrane potential assay

After NP treatment, mitochondrial membrane potential (ΔΨm, MMP) was analyzed using MitoTracker® Red CMXRos (ThermoFisher), according to manufacturer’s recommendations. After 45 min treatment with MitoTracker Red CMXRos reagent, spheroids were fixed with 4% PFA for 1 h at RT, washed with PBS and mounted on glass slides. Images were acquired with fluorescence microscope (Olympus BX60) and red fluorescence intensity was quantified by ImageJ software (https://imagej.nih.gov/ij/index.html, NIH).

### Lactate dehydrogenase (LDH) release assay

LDH release was determined by colorimetric CytoTox 96 Cytotoxicity Assay kit (Promega). As positive control, spheroids were treated with 1% TX-100 for 30 min. After NP treatments, 20 μL of supernatants were transferred to 96-well plates followed by the addition of 20 μL of substrate solution. After 30 min of incubation in the dark at RT, 20 μL of stop solution was added to each sample. Color development was proportional to the number of cells with disruption of plasma membrane. Absorbance was measured at 490 nm. For evaluation of eventual colorimetric interference, NP diluted in culture medium were incubated with LDH positive control and substrate according to manufacturer’s instructions.

### Scanning electron microscopy (SEM)

Spheroids were fixed with 2.5% glutaraldehyde for 1 h at RT. After three washing steps with PBS, samples were postfixed with 1% osmium tetroxide for 90 min in the dark, followed by three washing steps with 0.1 M cacodilate buffer (pH 7.4) and distilled water. Samples were gradually dehydrated, dried at the critical-point and finally vaporized with platinum. Photomicrographs were obtained at 3 kV in a SEM Helios Nanolab 650 (FEI, ThermoFisher).

### RNA extraction and quantitative real-time polymerase chain reaction (qRT-PCR)

Total RNA was extracted from 3D cultures after NP treatments using Tripure isolation reagent (Roche) according to Chomczynski and Sacchi [109]. RNA quantity and purity was determined using NanoDrop 2000c (ThermoFisher). One microgram of RNA was reverse-transcribed using the M-MLV Promega Reverse Transcriptase (Promega) according to the manufacturer’s recommendations. The expression of genes was evaluated using specific TaqMan gene expression assays (ThermoFisher). qRT-PCRs were performed using a 7500 Fast Real Time system (Applied Biosystems). Relative mRNA expression was calculated using the 2^−ΔΔCt^ method [110]. The analyzed genes were: SOD1 (Hs00533490_m1), NF2L2 (Hs00975961_g1), NFR1 (Hs00602161_m1), GSTO1 (Hs02383465_s1), CLEC7A (Hs01902549) and SOD2 (Hs00167309_m1). β-actin (BrainSpheres) and 18S (LUHMES) were used as housekeeping gene.

### Analysis of multiple secreted mediators

Determination of cytokines, chemokines and growth factors secreted by 3D LUHMES and BrainSpheres cultures upon NP exposure was carried through Luminex (Austin TX, USA) xMAP magnetic technology for the following analytes: IL-1β, IL-1ra, IL-2, IL-4, IL-5, IL-6, IL-7, IL-8, IL-9, IL-10, IL-12 (p70), IL-13, IL-15, IL-17, eotaxin, bFGF, GCSF, GM-CSF, IFN-γ, IP-10, MCP-1 (MCAF), MIP-1α, MIP-1β, PDGF-BB, RANTES, TNFα and VEGF, and TGF-β1, TGF-β2 and TGF-β3. Analysis was performed following the manufacturer’s recommendations. Briefly, after calibration and validation of Bio-Plex Magpix (Bio-Rad), reagent reconstitution and standard curve preparation, magnetic beads were added to each well of the assay plate. Each step was preceded by washing steps using an automated Bio-Plex Pro wash station (Bio-Rad). Then, samples, standard and controls were added, followed by detection antibodies and streptavidin-PE. Finally, magnetic beads were re-suspended and read. The number of analytes detected in culture medium without spheres (background) was subtracted from the samples, allowing to access the protein levels secreted by cultures.

## Additional files

Additional file 1: Z-stack from RFP-LUHMES treated with PLA NP, demonstrating internalization of NP. (GIF 4019 kb)

Additional file 2: 3D reconstruction of additional file 1 demonstrating internalization of PLA NP in RFP-LUHMES model. (GIF 4152 kb)

Additional file 3: **Figure S1.** Flow cytometry analysis of RFP-LUHMES exposed to PLA NP. (A) Dot plots of 3D LUHMES exposed to 0.2, 2 and 20 μg/mL PLA NP. Wild type LUHMES were used to set up the gates. (B) and (C) image galleries generated by ImageStream X Marc II flow cytometer from untreated RFP-LUHMES and RFP-LUHMES treated with 20 μg/mL PLA respectively. Ch01 – phase contrast, Ch02 – green, Ch04 – red and Ch06- site scater. (D) Histogram of green fluorescence intensity in untreated RFP-LUHMES (red), RFP-LUHMES treated with 20 μg/mL PLA (green) and RFP-LUHMES treated with 20 μg/mL PLA and subsequently with 40 μg/mL of trypan blue (blue). Table shows the number of live cells gated in each sample and median of the fluorescence intensity in each sample. (TIF 1877 kb)

Additional file 4: **Figure S2.** Effect of NP on expression of genes related to ROS regulation in 3D LUHMES. Graphs showing the relative expression of *SOD1*, *NF2L2*, *NFR1, GSTO1* and *SOD2* after exposure to Au-SC (6 μg/mL), Au-PEG (20 μg/mL) and PLA-NP (20 μg/mL) for 72 h normalized to the expression of the genes in the untreated control spheroids. Data was collected from two independent experiments with total eight biological replicates and represents fold changes (FC ± SEM). Only four replicates were used for Au-SC treated samples. One-way ANOVA with Dunnett’s multiple comparisons post-test was used to analyze the statistical significance. (TIF 86 kb)

Additional file 5: **Figure S3.** Influence of NP on release of TGF-β isoforms in 3D LUHMES. Graphs show the levels of secreted TGF-β1, TGF-β2 and TGF-β3 after exposure to Au-SC (6 μg/mL), Au-PEG (20 μg/mL) and PLA-NP (20 μg/mL) for 24 and 72 h in comparison to the untreated control spheroids. Each experimental group corresponds to the analysis of three independent experiments with three replicates and represents mean (± SEM). (TIF 3028 kb)

## Data Availability

The datasets used and/or analyzed during the current study are available from the corresponding author on reasonable request.
