# Supplementary figures and images for: Suitability of 3D human brain spheroid models to distinguish toxic effects of gold and poly-lactic acid nanoparticles to assess biocompatibility for brain drug delivery
Source: Part Fibre Toxicol. 2019 Jun 3;16:22. doi: 10.1186/s12989-019-0307-3 (PMC6545685; doi:10.1186/s12989-019-0307-3)

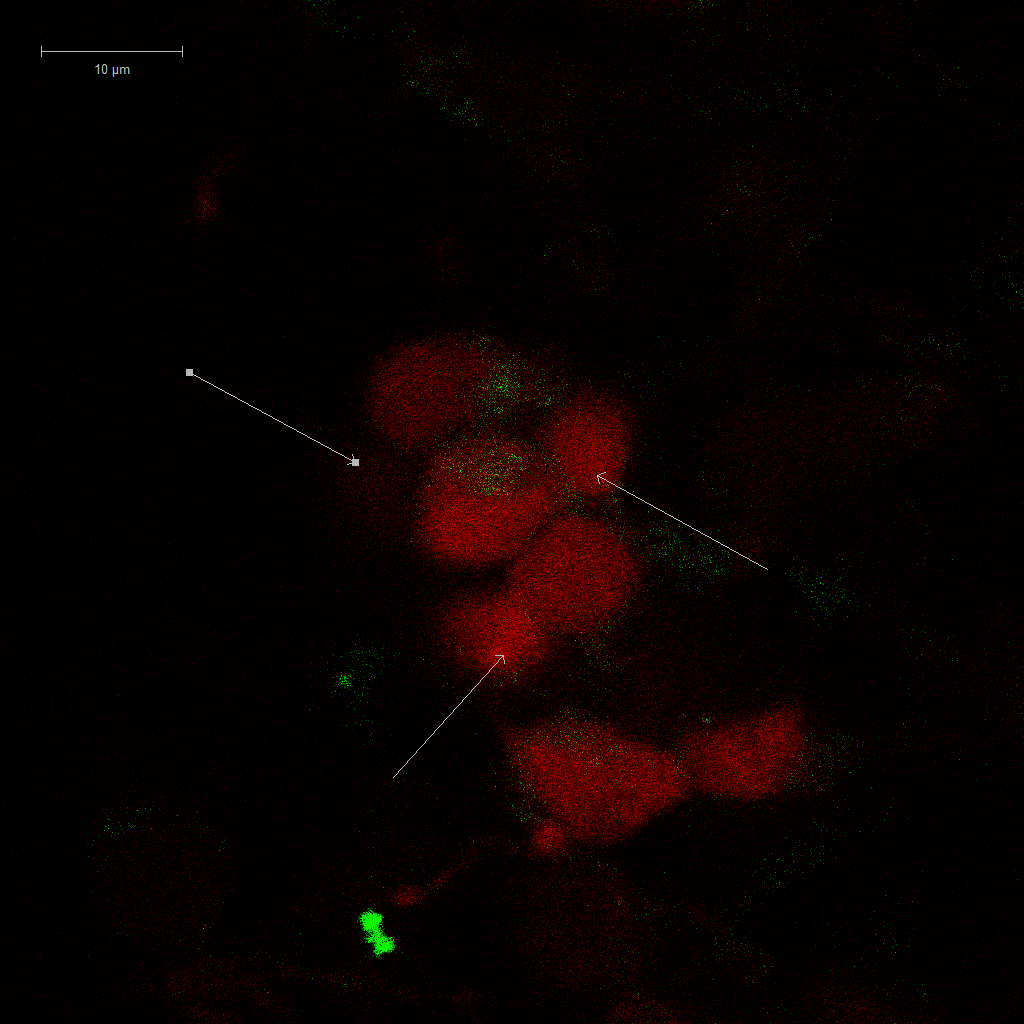

Supplement: Supplementary file 1 — Z-stack from RFP-LUHMES treated with PLA NP, demonstrating internalization of NP. (GIF 4019 kb) [file 12989_2019_307_MOESM1_ESM.gif]

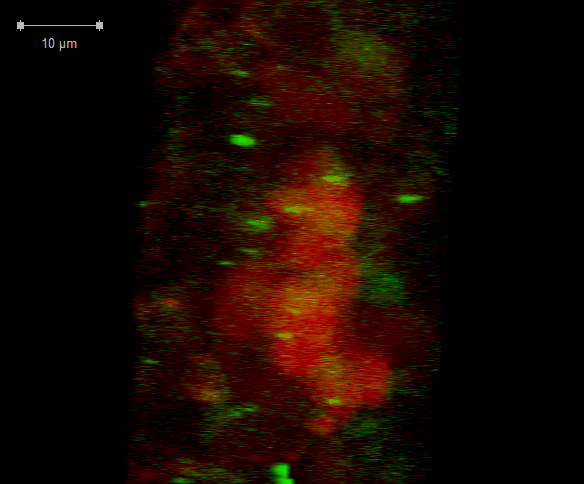

Supplement: Supplementary file 2 — 3D reconstruction of additional file 1 demonstrating internalization of PLA NP in RFP-LUHMES model. (GIF 4152 kb) [file 12989_2019_307_MOESM2_ESM.gif]

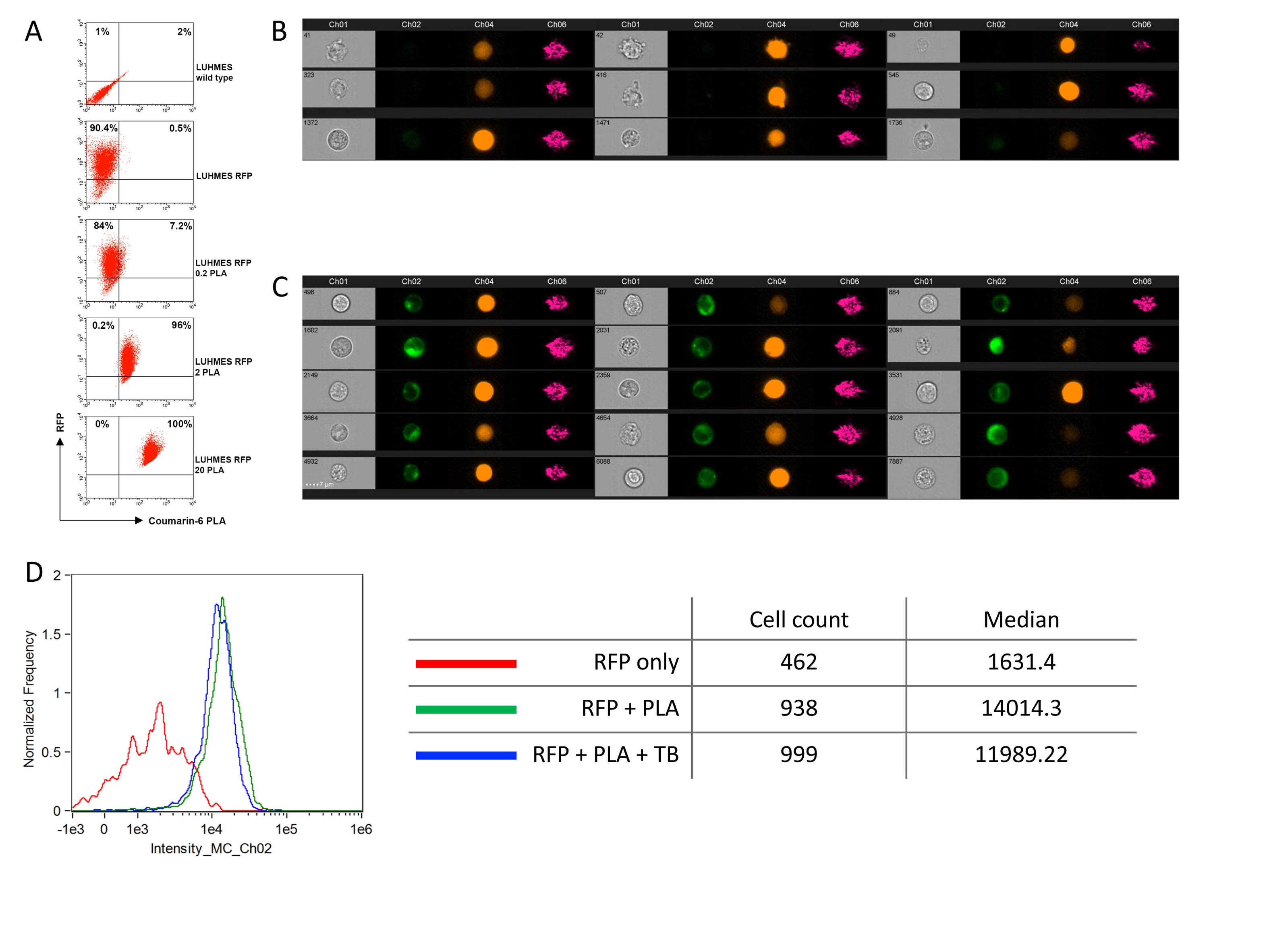

Supplement: Supplementary file 3 — Figure S1. Flow cytometry analysis of RFP-LUHMES exposed to PLA NP. (A) Dot plots of 3D LUHMES exposed to 0.2, 2 and 20 μg/mL PLA NP. Wild type LUHMES were used to set up the gates. (B) and (C) image galleries generated by ImageStream X Marc II flow cytometer from untreated RFP-LUHMES and RFP-LUHMES treated with 20 μg/mL PLA respectively. Ch01 – phase contrast, Ch02 – green, Ch04 – red and Ch06- site scater. (D) Histogram of green fluorescence intensity in untreated RFP-LUHMES (red), RFP-LUHMES treated with 20 μg/mL PLA (green) and RFP-LUHMES treated with 20 μg/mL PLA and subsequently with 40 μg/mL of trypan blue (blue). Table shows the number of live cells gated in each sample and median of the fluorescence intensity in each sample. (TIF 1877 kb) [file 12989_2019_307_MOESM3_ESM.tif]

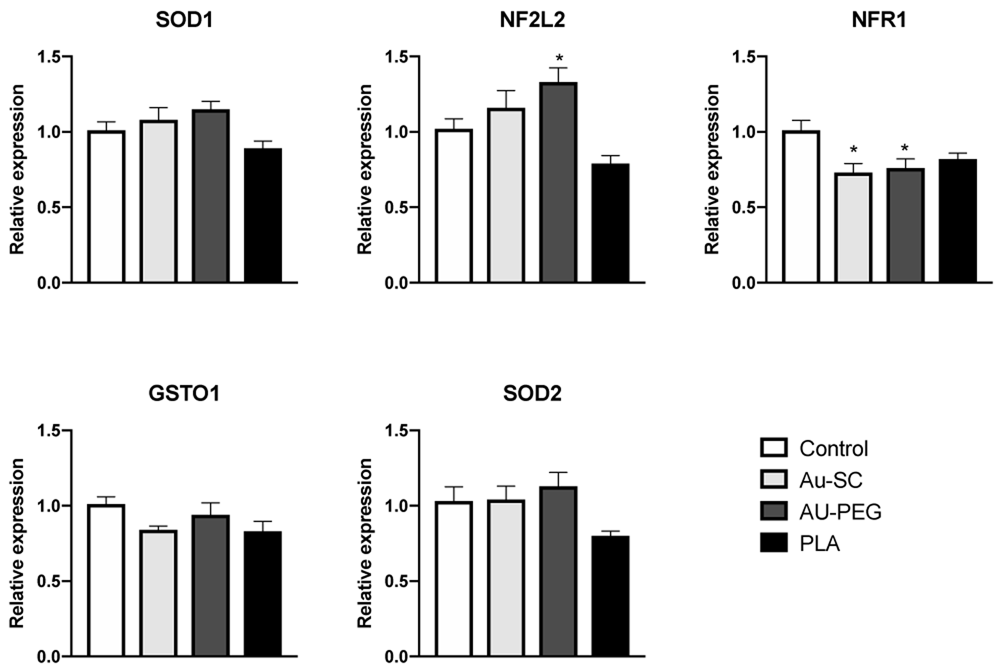

Supplement: Supplementary file 4 — Figure S2. Effect of NP on expression of genes related to ROS regulation in 3D LUHMES. Graphs showing the relative expression of SOD1, NF2L2, NFR1, GSTO1 and SOD2 after exposure to Au-SC (6 μg/mL), Au-PEG (20 μg/mL) and PLA-NP (20 μg/mL) for 72 h normalized to the expression of the genes in the untreated control spheroids. Data was collected from two independent experiments with total eight biological replicates and represents fold changes (FC ± SEM). Only four replicates were used for Au-SC treated samples. One-way ANOVA with Dunnett’s multiple comparisons post-test was used to analyze the statistical significance. (TIF 86 kb) [file 12989_2019_307_MOESM4_ESM.tif]

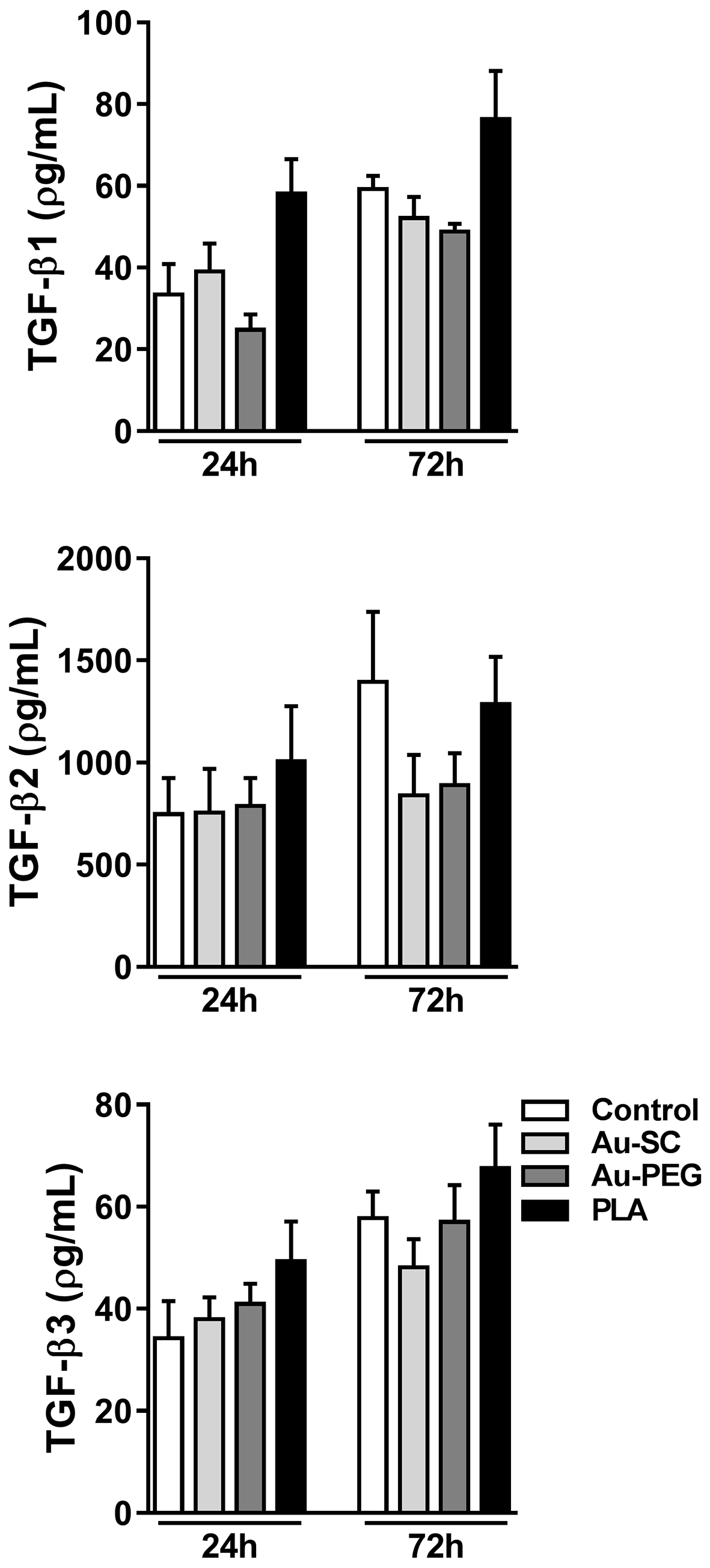

Supplement: Supplementary file 5 — Figure S3. Influence of NP on release of TGF-β isoforms in 3D LUHMES. Graphs show the levels of secreted TGF-β1, TGF-β2 and TGF-β3 after exposure to Au-SC (6 μg/mL), Au-PEG (20 μg/mL) and PLA-NP (20 μg/mL) for 24 and 72 h in comparison to the untreated control spheroids. Each experimental group corresponds to the analysis of three independent experiments with three replicates and represents mean (± SEM). (TIF 3028 kb) [file 12989_2019_307_MOESM5_ESM.tif]
